# CD157: From Myeloid Cell Differentiation Marker to Therapeutic Target in Acute Myeloid Leukemia

**DOI:** 10.3390/cells8121580

**Published:** 2019-12-05

**Authors:** Yuliya Yakymiv, Stefania Augeri, Giulia Fissolo, Silvia Peola, Cristiano Bracci, Monica Binaschi, Daniela Bellarosa, Andrea Pellacani, Enza Ferrero, Erika Ortolan, Ada Funaro

**Affiliations:** 1 Laboratory of Immunogenetics, Department of Medical Sciences, University of Torino, 10126 Torino, Italy; yuliya.yakymiv@edu.unito.it (Y.Y.); stefania.augeri@unito.it (S.A.); giulia.fissolo@edu.unito.it (G.F.); silvia.peola@unito.it (S.P.); cristiano.bracci@edu.unito.it (C.B.); enza.ferrero@unito.it (E.F.); erika.ortolan@unito.it (E.O.); 2 Department of Experimental and Translational Oncology, Menarini Ricerche S.p.A, 00071 Pomezia, Rome, Italy; mbinaschi@menarini-ricerche.it (M.B.); dbellarosa@menarini-ricerche.it (D.B.); 3 Menarini Ricerche S.p.A, 50131 Florence, Italy; apellacani@menarini-ricerche.it

**Keywords:** CD157/BST-1, myeloid cells, stem cells, cyclic ADPR, cell adhesion, acute myeloid leukemia, therapeutic defucosylated monoclonal antibody, MEN1112/OBT357

## Abstract

Human CD157/BST-1 and CD38 are dual receptor-enzymes derived by gene duplication that belong to the ADP ribosyl cyclase gene family. First identified over 30 years ago as Mo5 myeloid differentiation antigen and 10 years later as Bone Marrow Stromal Cell Antigen 1 (BST-1), CD157 proved not to be restricted to the myeloid compartment and to have a diversified functional repertoire ranging from immunity to cancer and metabolism. Despite being a NAD^+^-metabolizing ectoenzyme anchored to the cell surface through a glycosylphosphatidylinositol moiety, the functional significance of human CD157 as an enzyme remains unclear, while its receptor role emerged from its discovery and has been clearly delineated with the identification of its high affinity binding to fibronectin. The aim of this review is to provide an overview of the immunoregulatory functions of human CD157/BST-1 in physiological and pathological conditions. We then focus on CD157 expression in hematological tumors highlighting its emerging role in the interaction between acute myeloid leukemia and extracellular matrix proteins and its potential utility for monoclonal antibody targeted therapy in this disease.

## 1. Introduction

**Figure 1:**
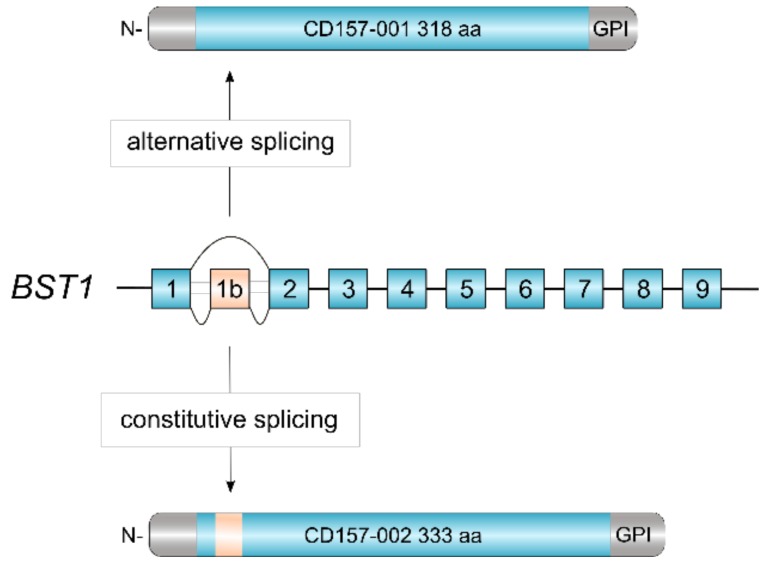
Alternative splicing of human *BST1*. The revised structure of human *BST1* consisting of 10 exons [6]. Skipping of exon 1b by alternative splicing yields the canonical CD157-001 isoform of 318 aa whereas inclusion of exon 1b adds 15 aa in-frame to the polypeptide, yielding the CD157-002 isoform of 333 aa.

CD157 is a glycosylphosphatidylinositol (GPI)-anchored glycoprotein, discovered over three decades ago and originally designated as Mo5 myeloid cell differentiation marker [1]. Subsequently, in the VI Workshop on Leukocyte Differentiation Antigens, Mo5 and the molecule recognized by the RF3 anti-Bone Marrow Stromal Cell Antigen 1 (BST-1) monoclonal antibody were grouped together and designated as CD157 [2]. The human *BST1* gene maps to chromosome 4p15.32, adjacent to its paralog *CD38* with which it forms part of the ADP ribosyl cyclase (ARC) gene family [3]. Comparative gene analysis revealed a striking exon-intron structural similarity between *BST1* and *CD38*, indicating that the two genes evolved by duplication from an ancestral gene before the divergence of humans and rodents [4]. Following duplication, the two genes went their separate ways and diverged in structure and sequence under the influence of mutation, selection and drift [3]. Human *BST1* was cloned in 1994 and one *BST1* transcript was identified which encoded the canonical CD157/BST-1 protein of 318 amino acids [5]. Recently, our laboratory described a second CD157/BST-1 transcript which encompasses an additional exon interposed between exons 1 and 2 of the *BST1* gene (Figure 1). This 10-exon transcript encodes a protein of 333 amino acids, named CD157-002. This serendipitous finding revealed that human CD157 is so far the only member of the ARC gene family regulated by alternative splicing. The two transcripts appear to be generally co-expressed, although the CD157-001 transcript is usually far more highly expressed [6].

Human *BST1* variants have been described with four single-nucleotide polymorphisms (SNPs) identified as risk factors for sporadic late-onset Parkinson’s disease (PD) in a Japanese GWA study [7], and in the Northern Han Chinese population [8,9], while this finding remains controversial in the European population [10,11]. Moreover, three possible risk SNPs for autism spectrum disorders (ASD) were identified in a Japanese population [12]. However, these variants were not found significantly associated with ASD or with the severity of the disease in the Han Chinese population [13]. It is conceivable that clinical and genetic heterogeneity of ASD and PD and the complexity of their inheritance patterns may justify variable distribution of these SNPs in different ethnic populations. Although the causal link between the *BST1* SNP and brain diseases remains unclear, functional implications of CD157 in the pathophysiology of several neurologic disorders are supported by the observation of partially deleted *BST1* and *CD38* genes in an ASD patient [14] and by the impaired social behaviors associated with anxiety and depression occurring in *Bst1* knockout mice [15]. Since CD157 is expressed in mouse brain, especially during embryonic development, it has been speculated that it might be involved in the processes of neuronal development that relates to neurologic disorders such as PD and ASD [16].

## 2. CD157 Protein Structure and Tissue Distribution in Health and Disease

The CD157 protein contains four predicted *N*-linked glycosylation sites in the extracellular region [17] facilitating the folding of the nascent polypeptide chain into a conformation fitting intracellular transport and enzymatic activity. The molecular weight of CD157 ranges between 42–50 kDa, according to its heterogeneous glycosylation patterns [18,19]. Dimeric forms of CD157 were detected when the protein was exogenously expressed at high epitope density in MCA102 and CHO fibroblasts [20].

In addition to the GPI-anchored form, a soluble form of CD157 is detected in serum and other biological fluids. Very high levels of soluble CD157 were reported in the sera of patients with rheumatoid arthritis compared to healthy controls [21]. Elevated soluble CD157 was also found in pleural effusions from patients with malignant pleural mesothelioma compared to patients with other cancer types or benign pathologies [22]. Recently, the concentration of soluble CD157 was found to be significantly increased in pleural fluid obtained from tuberculous pleurisy patients with respect to patients with pneumonia or lung cancer [23].

CD157 can be shed either as a soluble protein, generated by proteolytic cleavage of the membrane-bound form, or as an exosome-anchored protein. CD157 was found to be expressed by exosomes released by myeloid-derived suppressor cells [24] and by mesothelioma cells, both in vitro [25] and in vivo [22]. The functional role of soluble CD157 remains to be defined.

As stated in the pioneering studies performed by Todd et al. in the 1980s, in the human hematopoietic system CD157 is prevalently expressed by cells of the myelomonocytic lineage [1,26]. In normal hematopoietic cells, CD157 is expressed at low levels throughout neutrophil maturation, its expression level progressively increasing at the later stages of maturation, from the promyelocytic stage onwards [27]. CD157 is also expressed in myeloid-derived suppressor cells, which expand during chronic and acute inflammatory conditions [28] and in patients with myeloid hematological malignancies [29]. CD157 is not expressed in early CD34^+^ monocytic precursors but it becomes clearly expressed in early stage of maturation characterized by low expression of CD36, and strongly expressed in the more mature CD36^high^ monocytic cells [30] and in macrophages [31]. However, increasing evidence shows that CD157 actually has a broader pattern of expression than previously thought. Indeed, in addition to bone marrow stromal cells, vascular endothelium [5], and circulating endothelial cells [32], CD157 is expressed in several other cell types and tissues of both lymphoid and nonlymphoid origin [33]. The general picture that emerges from studies performed in humans and mice indicates that CD157 expression is not restricted to the myeloid compartment but extends to tissues of different origins, such as gut [34], lung [35], eye [36], blood vascular system [37], and brain [16]. Of note, its expression frequently marks cell stemness [38,39,40].

In hematological malignancies, low expression of CD157 was reported in the HL-60 acute promyelocytic leukemia cell line [41]; however, no data are available on the expression of CD157 in patients with this leukemia. CD157 expression in acute myeloid leukemia is described below (Section 7). CD157 has been reported to play a role in disease progression in certain malignancies by regulating cell invasion and metastasis. Our group reported that CD157 is expressed in >90% of primary serous epithelial ovarian cancer [42] where high CD157 expression correlates with increased tumor aggressiveness [43], promotes epithelial-to-mesenchymal transition [44] and is an independent prognostic factor for overall survival [45]. CD157 is also expressed in >85% of malignant pleural mesothelioma, where high CD157 levels are associated in vitro with enhanced tumorigenic potential and with reduced sensitivity to platinum-based chemotherapy, especially in the biphasic histotype [45]. In several in vitro cancer models, CD157 knockdown was consistently found to hamper cell migration, adhesion and tumorigenic potential [33].

Although for a long time CD157 has remained the neglected member of the ARC family, in recent years, it has gained interest as the functional repertoire of CD157 has greatly expanded from immunity to cancer and metabolism, with a common thread of cell adhesion/migration and ‘stemness’ connecting all functions [16,37,38,39,40].

## 3. CD157 Enzymatic Activity

Human CD157 has generated much less interest as an enzyme than as a receptor, perhaps because its catalytic activity is rather poor [46], or because its original identification as a bone marrow stromal antigen supporting the growth of murine immature B cells in vitro drew immediate attention to its receptor activities [47]. However, subsequent experimental evidence demonstrated that human CD157 can convert β-NAD^+^ mostly to ADP ribose with trace amounts of cyclic ADP ribose (cADPR), indicating the presence of ADP-ribosyl cyclase, NAD glycohydrolase, and possibly cADPR hydrolase activities [48,49]. It is speculated that NAD^+^ might be released from lysed intracellular stores under conditions of cell stress or inflammatory stimuli. Convincing evidence for a biological role of CD157 as an ectoenzyme arises from at least two experimental mouse models that are reliant upon cADPR generated by murine CD157, even though it may be produced in hormone-like concentrations. First, subnanomolar concentrations of extracellular cADPR are generated by CD157^+^ hemopoietic mesenchymal stromal cells; although insufficient to perturb intracellular Ca^2+^ concentrations, the amount of cADPR generated was sufficient to exert a paracrine stimulatory effect on proliferation of hemopoietic progenitors during long-term culture [50]. Second, in calorie-restricted mice, cADPR was produced by highly CD157-positive Paneth cells and triggered intestinal stem cells to switch on maintenance rather than differentiation programs [40].

Unlike canonical CD157, CD157-002 proved to be devoid of NAD glycohydrolase activity in in vitro experiments, despite the two proteins having similar structural features, cell membrane localization, and specific antibody binding. The CD157 isoforms could also not be distinguished in their ability to act as adhesion proteins and signaling molecules [6]. Whether or not CD157 is involved in metabolic regulation in other tissues as well remains to be elucidated. To date, the biological role of CD157 enzymatic activity in humans remains enigmatic.

## 4. CD157 Receptor Activity

The capacity of CD157 to transduce intracellular signals in myeloid cells emerged long before it became a member of the ARC family and its enzymatic nature was established [5]. As they were dealing with an orphan receptor with an unknown non-substrate ligand, immunologists investigating the functions of CD157 took advantage of polyclonal and monoclonal antibodies (mAb) as surrogates of the natural ligand(s). Using this approach, it emerged that crosslinking CD157 by means of a specific polyclonal antibody induced tyrosine phosphorylation of a 130-kDa protein (p130) in the human myeloid cell lines U937 and THP1 [31], subsequently identified as focal adhesion kinase (FAK) [51]. Instead incubation of neutrophils with anti-CD157 mAbs inhibited their phagocytic activity and blocked the NADPH oxidase-catalyzed generation of superoxide in U937 cells [52]. Our group demonstrated that CD157 ligation by specific mAbs promotes neutrophil polarization [53], pointing to a key role of CD157 in orchestrating leukocyte trafficking. Indeed, in neutrophils and monocytes CD157 modulates adhesion to the extracellular matrix (ECM), motility and transendothelial migration [53,54] through the activation of the MAPK/ERK1/2 and PI3K/Akt signaling pathways [43]. Engagement of CD157 in human umbilical vein endothelial cells increases intracellular Ca^2+^ concentration, which is instrumental for cytoskeletal actin reorganization which is thought to enhance interendothelial gap formation favoring leukocyte transmigration [55]. The contribution of CD157 to epithelial ovarian cancer progression relies on its ability to switch on a differentiation program that allows cancer cells to overlook the rules of epithelial tissue architecture and acquire mesenchymal features, required to advance in their malignant progression [44]. In malignant mesothelioma, CD157 exerts its pro-tumorigenic effects through the activation of the PI3K/AKT/mTOR pathway [45].

**Figure 2:**
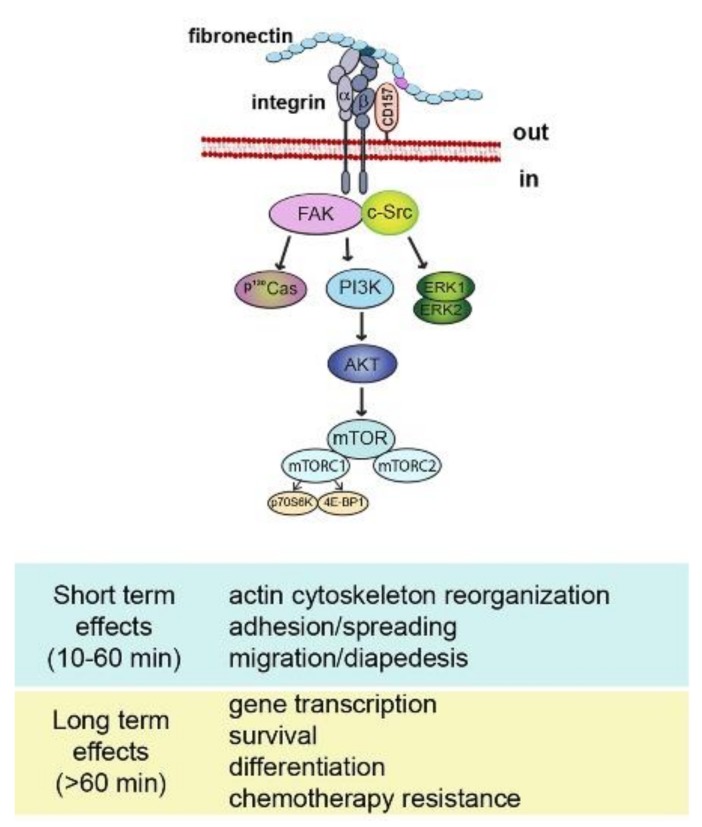
Schematic representation of CD157-mediated intracellular signals. Binding of CD157 to fibronectin is instrumental to form a multimolecular complex with integrins and to promote the assembly of a network of interconnected intracellular signals eliciting short term and long term effects.

Despite the compelling evidence of its receptor role, CD157 lacks intracellular domains, therefore, it is structurally unfit to transduce signals by itself. However, like many other GPI-anchored proteins, CD157 exploits its dynamic behavior in terms of diffusion, organization, and interactions with other membrane-spanning proteins to establish functional and structural cross-talk with transmembrane receptors. In particular, ligation by specific mAbs to CD157 expressed in leukocytes promotes its association with CD29 (β1 integrin) and CD18 (β2 integrin) favoring the organization of multimolecular complexes mediating outside-in signal transduction that regulates cell adhesion and motility [56,57] and promotes migration, osteogenic differentiation [58] and self-renewal in human mesenchymal cells [39] (Figure 2).

A protective role against *M. tuberculosis* infection in mouse has been recently attributed to CD157. To accomplish this function, CD157 enhances the compartmentalization of TLR2 and PKCzeta, and selectively drives ROS production [23]. However, the downstream signaling pathway underpinning CD157-mediated ROS production remains unknown.

## 5. CD157 and Its Nonsubstrate Ligands

The identification of the key role of CD157 in cell adhesion to the ECM provided valuable insights into the biological mechanism responsible for the receptor functions of CD157 in physiological conditions and in selected pathological contexts. Using solid-phase immunoenzymatic and Surface Plasmon Resonance assays, we demonstrated that CD157 binds with high affinity to the N-terminal (HBD1) and C-terminal (HBD2) heparin-binding domains of fibronectin, as well as to the HBD of collagen I, fibrinogen, and laminin-1 [59]. This finding turned out to be crucial for understanding how CD157 acquires receptor functions. Indeed, fibronectin (as other ECM proteins) by virtue of its ordered domain organization, simultaneously interacts with multiple ligands - in particular, integrins and CD157—thus favoring the organization of multimolecular complexes mainly located in plasma membrane microdomains enriched with signaling elements. These protein complexes are instrumental for the delivery of optimal signals that regulate many cellular functions, including cell adhesion and migration. Consistent with this assumption, genetic knockdown of CD157 led to attenuated fibronectin-mediated activation of FAK, Src, and Akt tyrosine kinases and eventually affected cell adhesion and spreading [59].

In human mesenchymal stem cells CD157 interacts with integrin β1 or β2 thus generating a protein complex that acts as a receptor for the SCRG1(scrapie responsive gene 1) protein. SCRG1 is a soluble protein preferentially expressed in the central nervous system, associated with neurodegenerative changes observed in transmissible spongiform encephalopathies. The SCRG1/BST-1/integrin cross-talk maintains mesenchymal stem cell self-renewal and multipotency and promotes the migration of human bone marrow-derived mesenchymal stem cells during bone regeneration through the activation of the FAK/PI3K/Akt signaling pathway [58]. In mouse macrophages, the SCRG1/BST-1/integrin interaction has been reported to activate MAPK/ERK1/2 pathway thereby suppressing LPS-induced CCL22 (C-C Motif Chemokine Ligand 22) production [60]. However, the functional significance of this interaction in vivo remains to be clarified.

The observation that CD157 expressed in bone marrow stromal cells supports the growth of pre-B cells fostered the hypothesis of the existence of a membrane ligand expressed by pre-B cells, but this assumption has never been substantiated by experimental evidence [61].

## 6. Role of CD157 in the Innate and Adaptive Immune Response

In the mouse model, several studies demonstrated that CD157 is transiently expressed by both B and T cell progenitors undergoing gene rearrangement of the antigen receptor [62], and that CD157 is implicated in early B and T cell growth and development [63,64]. Further data pointing to an important role for CD157 in B lymphocyte development and migration is that the *Bst1* gene has been identified as a target of the transcription factor Pax5, the B-cell identity factor, in murine pro-B cells [65]. PAX5 activation is implicated in the control of signal transduction from the pre-BCR and BCR, which constitute important checkpoints in B cell development, adding supportive evidence that murine CD157 functions during rearrangement of antigen receptor genes [66]. The expression pattern of CD157 in the rat differs from that in the mouse and rat CD157 is apparently not implicated in T or B cell development [67]. No defective B cell development was associated with *Bst1* gene deletion, while humoral T-independent immune responses and the thymus-dependent antigen-specific mucosal immune responses after oral immunization were found to be deficient in *Bst1* knockout mice [68]. There is currently no evidence that human CD157 is involved in B or T cell development.

The findings that human CD157 is constitutively expressed in neutrophils, monocytes and vascular endothelial cells (mainly at interendothelial junctions) [26] were highly suggestive of CD157′s implication in the control of leukocyte trafficking. Our group has dedicated considerable effort to elucidate the role of human CD157 in the innate and adaptive immune response, demonstrating that CD157 plays a pivotal role in the control of neutrophil and monocyte trafficking. Our first studies performed using CD157-specific agonist (or blocking) antibodies as a surrogate for the ligand, showed that CD157 cross-linking in neutrophils elicited Ca^2+^ currents both of extracellular and intracellular origin, apparently not mediated by cADPR nor ADPR [53]. The amplitude of the signal proved dependent on the extent of cross-linking, corroborating the assumption that the redistribution of the protein on the membrane is crucial for the generation of signaling-competent microdomains. Moreover, CD157 clustering by specific antibodies regulates neutrophil and monocyte adhesion to ECM proteins, motility and transendothelial migration [53,54]. These biological functions rely on the formation of multimolecular complexes favoring structural juxtaposition of CD157 with β1- and β2-integrin, an essential requirement for conferring CD157 receptor functions [57].

The discovery that CD157 binds with high affinity to the heparin binding domains located within fibronectin or other components of the ECM, finally assigned to CD157 the designation of adhesion protein, and unveiled how CD157 accomplishes its receptor functions in physiological conditions (e.g., leukocyte trafficking) and in specific pathological contexts (e.g., inflammatory diseases and cancer) [42,45,59,69].

## 7. CD157 in Hematologic Malignancies

CD157 presents a restricted pattern of expression in hematological malignancies, being present only in B-cell precursors acute lymphoblastic leukemia and acute myeloid leukemia.

### 7.1. B-Cell Precursor Acute Lymphoblastic Leukemia

In B-cell precursor acute lymphoblastic leukemia, CD157 has been proposed as a diagnostic marker for disease monitoring. Indeed, Cell Surface Capture technology highlighted that high CD157 expression levels discriminates tumor cells from normal B cell populations that are CD157-negative, and may serve as an additional marker to improve residual leukemia cell detection and quantification at early treatment time points [70].

### 7.2. Acute Myeloid Leukemia

Acute myeloid leukemia (AML) is a clonal disorder of hematopoietic stem and progenitor cells that have lost the ability to differentiate into functional granulocytes or monocytes. It is characterized by accumulation of undifferentiated leukemia blasts showing cytogenetic and molecular abnormalities that causes alterations in cell self-renewal, proliferation, and differentiation. AML is the most common acute leukemia among adults, whose incidence increases with age with a 5-year overall survival rate of 40% for patients less than 60 years of age and only 10% for elderly patients above the age of 60 [71]. A majority of the patients with newly diagnosed AML achieve complete remission with intensive chemotherapy. However, approximately 60%–80% of patients relapse after frontline therapy within 3 years after diagnosis [72].

**Figure 3:**
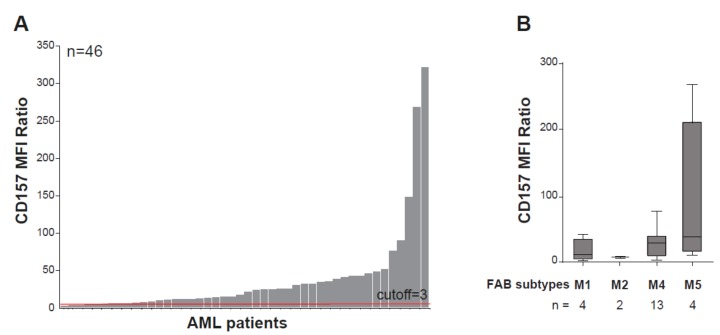
CD157 expression in acute myeloid leukemia. (**A**) Flow cytometry analysis of CD157 expression performed with the PE-labeled SY11B5 monoclonal antibody, in 46 primary acute myeloid leukemia (AML) samples at diagnosis. Leukemic blasts were gated by using standard side scatter (SSC) low CD45dim. (**B**) CD157 expression intensity in FAB AML subtypes in 23 bone marrow samples from patients with AML for which the FAB classification was available. Fluorescence was determined using a FACS Canto flow cytometer and analysed with FlowJo software (FlowJo, LLC). 10,000 events were analyzed for each sample. Geometric mean fluorescence intensity (MFI) values of CD157 were normalized to the MFI of the normal lymphocytes within each sample, which were negative for CD157. A total of 46 samples’ MFI ratios (MFI blast/MFI lymphocyte) were subsequently analyzed for CD157 expression intensity. Samples with CD157 MFI ratio ≥ 3.0 were regarded as positive.

CD157 is expressed in 97% of AML patients at the time of primary diagnosis, regardless of the genetic profile, with remarkable inter-patient heterogeneity in expression levels (Figure 3A). Parallel to its high expression during monocytic cell differentiation, the highest expression of CD157 is associated with French American British (FAB) M4 and M5 (e.g., myelomonocytic and myelocytic leukemia) AML subtypes [17,73] (Figure 3B). In a small cohort of patients with AML, a correlation has been reported between high CD157 expression levels and the adverse prognosis group of patients according to the European Leukemia Net (ELN) classification-2017 [74], but not with *NPM1* and *FLT3-ITD* mutational status [73]. Comparison of CD157 surface expression by flow cytometry in paired diagnosis and relapse AML samples showed it was stably expressed [73], suggesting that it may be a helpful marker for minimal residual disease detection. Albeit at lower extent than in bulk AML blasts, CD157 was also found to be expressed in CD34^+^CD38^−^ leukemia-initiating cells [73], characterized by long-term repopulating potential, ability to propagate and maintain the AML phenotype, and believed to be the main cause for AML relapse [75].

In recent years, the bone marrow microenvironment has been recognized to be a dynamic and complex living tissue that, in addition to important homeostatic roles in hematopoiesis, can aid and abet neoplastic disease processes [76]. Accumulating evidence showed that the cross-talk between leukemic cells and both cellular and extracellular stromal elements in the bone marrow niche greatly influences leukemia progression and response to therapy [77]. Thus, deciphering these interactions has the potential to lead to the development of novel treatment strategies to overcome acquired drug resistance and prevent relapse after therapy [78,79].

In the past decades, various cell types were implicated for their roles in promoting leukemia maintenance, including perivascular stromal cells, endothelial cells, macrophages, fibroblastic CXCL12-positive reticular cells [77,80], all of which express CD157 [55,81,82,83]. These observations provided the rationale to address the biological function of CD157 in AML. Using a genetic approach, we got preliminary evidence that CD157 has a role in the interaction between leukemic blasts and ECM proteins. Indeed, shRNA knock-down of CD157 reduced THP1 and U937 AML cell adhesion to fibronectin, collagen type I, and fibrinogen, but not to vitronectin, which is not a CD157 ligand [59] (Y.Y., personal observation). Adhesion to fibronectin protects AML blasts from chemotherapy-induced cytotoxicity, an effect known as cell adhesion-mediated drug resistance [84]. Of note, the fibronectin-mediated protective effect against cytosine arabinoside treatment proved to be stronger in CD157-positive than in CD157-negative U937 cells (S.A., personal observation), suggesting that CD157 has a role in facilitating leukemia cell interactions with extracellular matrix components and modulates the sensitivity of AML cells to chemotherapy, at least in vitro.

**Figure 4:**
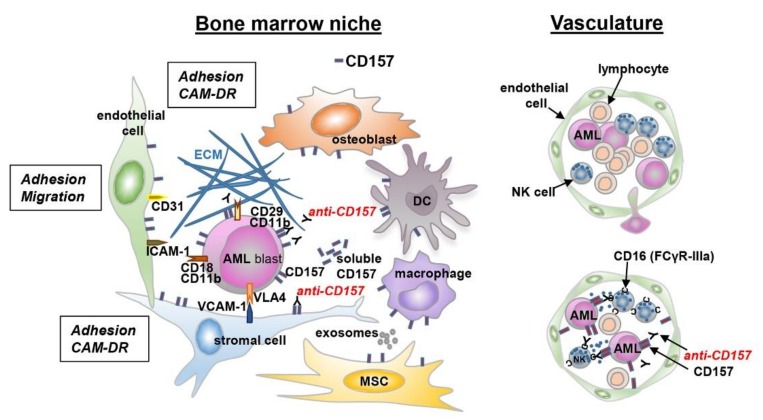
Schematic representation of the interaction between leukemic cells and bone marrow niche. A schematic view of the tumor microenvironment components is presented. AML cells are surrounded by a complex microenvironment composed of extracellular matrix (ECM) proteins and several cell types, including bone marrow stromal cells (endothelial cells, mesenchymal stromal cells, dendritic cells, macrophages, and immune cells). The cross-talk between AML cells and bone marrow stromal cells is regulated by different mechanisms: cell-to-cell adhesion between tumor cells and ECM components, or bone marrow stromal cells and soluble factors. These interactions activate several signaling pathways protecting tumor cells from chemotherapy-mediated toxicity (CAM-DR). The expression of CD157 on different cell types is indicated.

These preliminary experimental data suggest that CD157 is critical for integrating adhesive signals from the environment, hinting to the potential clinical utility of CD157 as a therapeutic target in AML (Figure 4). Indeed, antibodies that target leukemia-niche interactions are exciting new tools that may be used to disrupt the molecular mechanisms that keep leukemic blasts and leukemic stem cells in their protective BM niche, making them susceptible to chemotherapy or immune attack.

## 8. CD157 As a Target for Therapy in AML

AML has been so far challenging to treat with antibody targeted immunotherapy, owing to the lack of leukemia-specific surface markers; indeed, antigens expressed by AML cells are usually shared by normal myeloid progenitors and differentiated myeloid cells. It is likely that there is no single optimal target for antigen-directed immunotherapy in a disease as heterogeneous as AML. Different immunotherapeutic modalities, including unconjugated, toxin-conjugated, and radio-conjugated antibodies as well as multivalent formats are currently under development. These antibodies target various antigens in AML, including CD33, CD38, CD123, and CLL-1 [85,86,87].

CD157 represents a promising candidate as therapeutic target in AML, since it is broadly expressed in leukemic cells from peripheral blood and from bone marrow, both at diagnosis and disease relapse [73], and is implicated in leukemia-bone marrow niche interactions, thus offering multiple levels of targeting.

Antibody-dependent cell cytotoxicity (ADCC) and antibody-dependent cell phagocytosis are key mechanisms of action exploited by many therapeutic antibodies to eliminate tumor cells. The efficacy of these mechanisms relies, at least in part, on low internalization rate of the target antigen [88]. The low internalization rate of CD157 once bound by specific antibodies hints to its suitability as target for ADCC.

So far, the use of unconjugated antibodies eliciting ADCC in AML has not been successful, despite sensitivity of AML cells to NK-mediated cytotoxicity. This was demonstrated both in vitro and in patients treated with adoptive immunotherapy exploiting alloreactive NK cells [89,90]. One of the reasons why this approach fails is likely the impaired function and reduced number of NK cells in AML patients. Experimental ex vivo data highlighting defects in lytic synapse formation between primary AML blasts and NK cells support this assumption [91].

MEN1112/OBT357, a defucosylated, humanized, CD157-specific, monoclonal IgG_1_ has been generated and designed by Oxford BioTherapeutics (Oxford, UK) with the aim of overcoming the NK impairment in AML. There is a general consensus that ADCC is modulated by the *N*-linked glycosylation in the Fc region of the antibody. In particular, removal of the fucose at the conserved *N*-glycosylation site Asn^297^ in the CH2 domain of the Fc fragment has been shown to increase IgG_1_ Fc binding affinity to the FcγRIIIa present on immune effector cells, such as NK cells, leading to enhanced ADCC activity [92]. Defucosylation improves antibody affinity also for the low-affinity FcγRIIIa variant, characterized by the presence of a phenylalanine (Phe) at the amino acid residue 158 (F158). The relevance of the polymorphism of the FcγRIIIa transmembrane protein in the clinical efficacy of therapeutic antibodies has been shown both for rituximab as well as for trastuzumab. As an example, patients with FcγRIIIa homozygous genotype Val/Val or FcγRIIIa heterozygous genotype Val/Phe received greater benefit from trastuzumab treatment than patients with homozygous genotype Phe/Phe [93].

Of note, preclinical and clinical data with different defucosylated antibodies (some of which received FDA approval for marketing) showed that this approach can generate safe and effective compounds [94]. Currently, three defucosylated antibodies (e.g., obinutuzumab, benralizumab, and mogamulizumab) are approved for different indications, and more than twenty additional defucosylated antibodies are in various stages of clinical development for the treatment of different diseases ranging from cancer to respiratory and autoimmune diseases.

MEN1112/OBT357 is characterized by high affinity for both the CD157 glycoprotein expressed by AML cells, through the Fab region, and for the FcγRIIIa receptor, through the Fc region, which warrant stronger ADCC in vitro, compared to its parental non-defucosylated analogue [73].

Although animal models have been the basic translational model in the preclinical setting to evaluate novel therapeutic anticancer drugs before clinical testing, they proved elusive for AML [95,96]. Indeed, AML in vivo mouse models fail to reproduce AML clonal heterogeneity, and murine effector cells number and function may substantially differ from those occurring in AML patients. Even in mice reconstituted with PBMCs from human healthy donors, the impairment of effector cells occurring in the disease is underestimated. Therefore, in AML, ex vivo studies are considered more informative than those in animal models to evaluate the therapeutic properties of monoclonal antibodies mainly acting through ADCC.

Accordingly, the ex vivo sensitivity assay was considered a pharmacologically and clinically appropriate model to evaluate the preclinical efficacy of MEN1112/OBT357. This antibody demonstrated potent ADCC activity in an autologous setting of primary AML blood and bone marrow samples compared to healthy donor blood samples, regardless of the FcγRIIIa polymorphism, and appreciable even at low target/effectors ratio. In this experimental condition, shedding of CD157 evaluated in patient sera was negligible and easily saturated at clinically relevant concentrations [97].

Moreover, safety and pharmacokinetics studies of MEN1112/OBT357 in non-human primates, the only informative animal model, showed an acceptable toxicological profile and established a half-life of approximately two weeks. In addition, the low immunogenicity of MEN1112/OBT357, although demonstrated in the non-clinical assessment, allowed a consistent exposure of non-human primates to the therapeutic antibody and, consequently, an appropriate evaluation of the non-clinical safety [97,98].

Altogether, the pre-clinical pharmacology, pharmacokinetics, immunogenicity and toxicology profiles supported the clinical development of MEN1112/OBT357. The ARMY-1 first-in-human clinical trial is currently ongoing, and consists in a dose escalation and in a cohort expansion phase, recruiting relapsed or refractory AML patients (NCT02353143).

Since more than 70% of AML express CD38, perhaps daratumumab (approved for use in multiple myeloma) can also be used in AML [87]. A phase II clinical trial is ongoing at MD Anderson Cancer Center evaluating the efficacy of daratumumab as a monotherapy for refractory/relapsed AML (NCT 03067571), while Ohio State University is evaluating its effectiveness in combination with donor leukocyte infusions for AML patients who have relapsed after allogeneic hematopoietic stem cell transplant (NCT 03537599). A study of Isatuximab (another anti-CD38 antibody [99]) recently opened a phase I/II trial for pediatric patients with refractory/relapsed AML and ALL for use with combination chemotherapy (NCT 03860844).

## 9. Conclusions and Perspectives

In this review, we have discussed the basic knowledge of human CD157 addressing its role in the immune system where, besides being a myeloid cell surface marker, it has a central role in the interaction between cells and the extracellular matrix. This interaction is instrumental for CD157 to exert its multiple receptor functions. In addition, we focused on acute myeloid leukemia where CD157 is consistently expressed throughout the course of the disease and, in some instances, it might directly contribute to leukemia maintenance and drug sensitivity. However, several unaddressed issues remain, including the relationship between enzymatic activities and receptor functions of CD157 in specific physiological and pathological scenarios. The emerging picture is that CD157 may represent a candidate target for novel therapeutic strategies in acute myeloid leukemia. However, like all therapeutic targets in advanced stages of development in AML, CD157 is not only expressed in leukemic cells but also in normal mature and immature myeloid cells and in vascular endothelial cells, possibly causing undesirable side-effects. Hence, optimization of the clinical use of CD157-specific immunotherapies could be challenging. Gemtuzumab ozogamicin is the best-studied therapeutic antibody in AML and much can be learned from its history of accelerated approval, withdrawal, and re-approval [100].
